# Effects of Sleep Deprivation on Performance during a Change Signal Task with Adaptive Dynamics

**DOI:** 10.3390/brainsci13071062

**Published:** 2023-07-12

**Authors:** Kimberly A. Honn, Megan B. Morris, Melinda L. Jackson, Hans P. A. Van Dongen, Glenn Gunzelmann

**Affiliations:** 1 Sleep and Performance Research Center & Department of Translational Medicine and Physiology, Washington State University, Spokane, WA 99202, USA; hvd@wsu.edu; 2 Air Force Research Laboratory, Wright Patterson Air Force Base, OH 45433, USA; 3 Turner Institute for Brain and Mental Health, School of Psychological Sciences, Monash University, Clayton, VIC 3800, Australia

**Keywords:** sleep loss, task performance, cognitive functioning, response time, response deadline, augmented cognition, executive functions, inter-individual differences

## Abstract

Augmented cognition, which refers to real-time modifications to a human–system interface to improve performance and includes dynamic task environments with automated adaptations, can serve to protect against performance impairment under challenging work conditions. However, the effectiveness of augmented cognition as a countermeasure for performance impairment due to sleep loss is unknown. Here, in a controlled laboratory study, an adaptive version of a Change Signal task was administered repeatedly to healthy adults randomized to 62 h of total sleep deprivation (TSD) or a rested control condition. In the computerized task, a left- or right-facing arrow was presented to start each trial. In a subset of trials, a second arrow facing the opposite direction was presented after a delay. Subjects were to respond within 1000 ms of the trial start by pressing the arrow key corresponding to the single arrow (*Go* trials) or to the second arrow when present (*Change* trials). The *Change Signal Delay* (*CSD*)—i.e., the delay between the appearance of the first and second arrows—was shortened following incorrect responses and lengthened following correct responses so that subsequent *Change* trials became easier or harder, respectively. The task featured two distinct *CSD* dynamics, which produced relatively stable low and high error rates when subjects were rested (*Low* and *High Error Likelihood* trials, respectively). During TSD, the *High Error Likelihood* trials produced the same, relatively high error rate, but the *Low Error Likelihood* trials produced a higher error rate than in the rested condition. Thus, sleep loss altered the effectiveness of the adaptive dynamics in the Change Signal task. A principal component analysis revealed that while subjects varied in their performance of the task along a single dominant dimension when rested, a second inter-individual differences dimension emerged during TSD. These findings suggest a need for further investigation of the interaction between augmented cognition approaches and sleep deprivation in order to determine whether and how augmented cognition can be relied upon as a countermeasure to performance impairment in operational settings with sleep loss.

## 1. Introduction

Sleep deprivation is increasingly common in our society, particularly in operational settings that support the 24/7 economy (e.g., transportation, emergency medicine, law enforcement, manufacturing). This is due to both job-related and personal factors, such as long work hours and night shifts, travel or commute time, extended social or leisure hours, and/or time demands such as from raising children, in addition to sleep disorders and other medical conditions that interfere with sleep [1]. Sleep deprivation and sleep deficiencies from sleep disorders and other medical conditions are prominently associated with vigilant attention deficits [2] as well as impairments to a range of other cognitive processes [3]. Yet, sleep deprivation-induced impairments are not global or universal. Some cognitive functions are generally resilient to sleep loss, such as working memory scanning efficiency [4] and semantic encoding [5]. By contrast, sleep deprivation produces particular vulnerabilities in tasks that require responding to unexpected or unannounced changes [6,7]. The impact of sleep deprivation overall increases the risk of errors and accidents [8]. The temporal dynamics of this risk increase are well understood [9,10], but the specific cognitive deficits involved remain unclear.

A variety of strategies and methods are used to reduce fatigue or limit risks associated with fatigue in operational settings [11,12]. Such strategies are typically either static features, including engineering approaches to make the environment safer (e.g., rumble strips), or reactive features, including alerts to potential hazards (e.g., lane deviation warnings) [13,14]. Alternative, proactive means of preventing fatigue-related errors may involve “smart” or dynamic adaptations to changes in performance or other signs of fatigue (e.g., through systems that monitor the physiological status of an individual through heart rate variability, eye gaze, or other metrics and modify workload or other task variables accordingly) [15]. However, the effectiveness of such safety features may be diminished when individuals are sleep-deprived [16,17,18].

One dynamic countermeasure approach, which may or may not help sleep-deprived people in mission-critical circumstances, involves augmented cognition [19]. A straightforward means to implementing this approach is based on systems with dynamic pacing in response to changes in task characteristics. These systems may guide the operator to work quickly when possible, particularly for time-sensitive tasks, but to slow down when accuracy is most imperative and/or when the task is more difficult. For example, dynamic pacing could guide a search and rescue mission to travel more quickly when flying over an open field, while making them slow down over a densely forested area. Similarly, for threat detection on land, on the sea floor, or in the air by means of unmanned systems using sonar and radar signals, data analysis could progress more quickly for sections with clear visibility and more slowly in environments where vision is obstructed or the setting is cluttered with distractions. Adaptive pacing may work well to optimize the speed and accuracy of performance under challenging circumstances when individuals are well-rested, but it is unknown if this approach continues to be effective when operators are fatigued due to sleep deprivation.

This knowledge gap is due, in part, to one of the hallmark effects of sleep deprivation, namely increased variability in performance [20]. That is, performance becomes unstable during sleep deprivation, meaning that errors made while fatigued are not simply a matter of slower performance [21] but rather a failure to maintain optimal performance consistently. Furthermore, other types of fatigue-related cognitive impairments may manifest, such as cognitive flexibility deficits [22] which may result in perseverative behavior. Thus, the effects of sleep deprivation on cognition and behavior are diverse [23]. By and large, studies have failed to identify the specific problems caused by sleep loss in particular tasks or cognitive functions [24,25]. As a result, little is known about how to best design task environments, system interventions, and augmented cognition approaches that would imbue resilience to the detrimental performance effects of sleep deprivation [26].

Here, we interrogated the effects of sleep deprivation in the adaptive task environment of the Change Signal task [27] as a proxy for an adaptive pacing-based augmented cognition approach to fatigue risk management. The Change Signal task was designed to maintain stable high or low performance accuracy levels in the face of changing operator performance by automatically adjusting the trial timing parameters based on previous trial accuracy. Using the Change Signal task in a controlled laboratory study, the present investigation sought to determine whether target performance accuracy levels managed by the task environment would remain stable when subjects are sleep-deprived.

## 2. Materials and Methods

### 2.1. Subjects

A total of *N* = 26 healthy adults (16 males, 10 females; ages 22–37 years, *M* ± *SD*: 25.2 ± 4.2 years) completed a 7-day/6-night in-laboratory study. Subjects were eligible for the study if aged 22–40 years, proficient English speakers, with normal sleep schedules and no relevant physical or mental health conditions, as confirmed through a telephone interview and two in-laboratory screening sessions. During the first in-laboratory screening session, prospective subjects completed a set of questionnaires regarding sleep, physical, and mental health, took an alcohol breathalyzer test, and had a urine drug screen and blood draw. During the second in-laboratory screening session, prospective subjects completed additional questionnaires and had a brief physical exam by a physician. On the first day of study participation, subjects repeated the breathalyzer (BACtrack S80, BACtrack, San Francisco, CA, USA) and urine drug screen prior to entering the laboratory.

All subjects were found to be physically and psychologically healthy, with no current medical or drug treatment (excluding oral contraceptives). They showed no significant abnormalities in blood and urine, and they were free of traces of drugs and alcohol. Subjects were found to have no current psychiatric illness and no presently clinically relevant history of psychiatric illness, no history of drug or alcohol abuse in the past year, and no history of methamphetamine abuse. They were not current smokers, had no history of moderate to severe brain injury and no history of a learning disability. Subjects had no sleep or circadian disorders, reported no previous adverse reaction to sleep deprivation, had regular bedtimes, habitually awoke between 06:00 and 09:00, had not traveled across time zones within one month of entering the study, and had no shift work within one month of entering the study. They were not vision impaired unless corrected to normal and were not pregnant.

During the week prior to the start of the laboratory study, subjects were asked to maintain their habitual sleep/wake schedule with strict regularity and abstain from napping, caffeine, drugs, and alcohol. They filled out a sleep/wake and performance diary, called a time-stamped voicemail box to report their bed/wake times, and wore a wrist actigraph continuously.

The study was approved by Washington State University’s Institutional Review Board (IRB). All subjects gave written, informed consent and were paid for their participation.

### 2.2. Experimental Design

Subjects participated in sets of up to four people, each with their own bedroom for sleeping and cognitive testing, and a shared common space for meals and free times. Each set was randomized to a well-rested control (WRC; *n* = 13) or a total sleep deprivation condition (TSD; *n* = 13).

Subjects entered the laboratory at 15:00 on day 1 and remained in the laboratory until the conclusion of the study at 22:00 on day 7. Light levels within the laboratory were dim (<100 lux) and temperatures were held constant (68–72 °F). Meals/snacks were served every 4 h of scheduled wakefulness, beginning at 16:30 on day 1. Outside of testing and sleeping times, subjects were free to chat with each other or the research assistants, read, watch movies, play board or card games, or engage in other low-key activities within the laboratory. Subjects were not allowed to exercise and had no access to live television, radio, internet, or telephone. There was around-the-clock supervision from laboratory staff to ensure the study protocol was followed, cognitive testing was performed under controlled conditions, and wakefulness was maintained outside of designated sleep opportunities.

Upon entering the study, subjects knew they would be randomized to the TSD or WRC condition (50% chance of each); they were informed of their assignment in the evening of day 2. The afternoon/evening of day 1 (Phase 0) was used for task training and adaptation to the laboratory environment. During the subsequent two nights and days of the study, all subjects spent 10 h time in bed (22:00–08:00) for baseline sleep. The period from 22:00 on day 1 through 22:00 on day 3 constituted the baseline period (Phase 1). Subjects in the TSD condition were then awake from 08:00 on day 3 through 22:00 on day 5 for a total of 62 h of continuous wakefulness. Subjects in the WRC condition had 10 h time in bed (22:00–08:00) each night during this interval. The period from 22:00 on day 3 through 22:00 on day 5 constituted the sleep deprivation/control period of the experiment (Phase 2). All subjects concluded the study with two nights and days with 10 h time in bed (22:00–08:00) for recovery sleep. The period from 22:00 on day 5 through 22:00 on day 7 constituted the recovery period (Phase 3).

**Figure 1:**
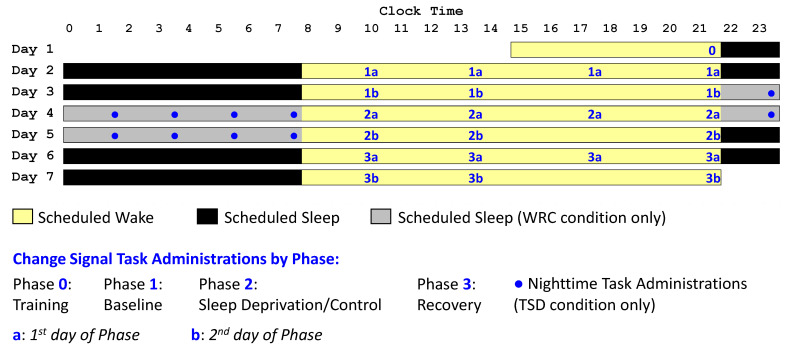
Study design. Subjects were in the laboratory for 7 days/6 nights. Time was spent awake (yellow), in bed for sleep (black), or in bed for sleep in the WRC condition only (gray). The Change Signal task (blue numbers when administered to all subjects; blue circles symbols when administered to TSD subjects only) was completed repeatedly throughout the study. The task training (Phase 0 administration) took place on day 1. The baseline period (Phase 1), sleep deprivation or control period (Phase 2), and recovery period (Phase 3) each included 7 daytime administrations of the Change Signal task. Phases 1–3 are subdivided to distinguish the first (a) and second (b) days of each Phase. During the sleep deprivation nights, there were additional Change Signal task administrations in the TSD condition only.

The Change Signal task was first administered at 21:30 on day 1. In line with previous studies using the Change Signal task, which provided a brief session of practice trials to ensure that subjects understood the task [28], the day 1 test bout was considered a training session and was omitted from analyses. The task was then administered at 10:00, 13:30, and 21:30 on each of the remaining days of the study. It was also administered at 17:30 on days 2, 4, and 6 (a different task, published elsewhere [29], was administered at 17:30 on days 3, 5, and 7). During the 62 h TSD period, the sleep deprivation group had additional Change Signal test bouts administered during the two nights of sleep deprivation (nights 3 and 4 of the study) at approximately 23:15, 01:15, 03:15, 05:15, and 07:15, making a total of 32 administrations in the TSD condition and 22 administrations in the WRC condition (see Figure 1).

### 2.3. Change Signal Task

The Change Signal task is a variation on the classic Stop Signal task [30]. In the Stop Signal task, subjects respond to a visual stimulus in a choice reaction time task. On a subset of trials, the visual stimulus is followed by a stop signal, such as an auditory tone. When a stop signal is presented, subjects are to inhibit their initially prepared response to the visual stimulus. A disadvantage of the Stop Signal task (and other go/no-go tasks) is that the inhibition process cannot be measured directly, as there is not meant to be a response following the stop signal [31]. In the Change Signal task, subjects also respond to a visual stimulus, such as an arrow pointing left or right. However, on a subset of trials, the visual stimulus is followed by a change signal, directing them to produce a different response, which is always the opposite of the initially prepared response. Thus, when a change signal is presented, subjects are to suppress their initial response and provide the alternate response instead. Compared to the Stop Signal task, the Change Signal task allows for assessment of the reaction time to the secondary stimulus and measurement of the effects of sleep deprivation on subjects’ ability to switch to an alternate response.

Each Change Signal task session took about 10 min to complete and consisted of 216 trials. At the beginning of the task, subjects were told that they would be shown an arrow pointing to the right or left, that the arrow would appear in one of two colors, and that they should press the matching arrow key on the keyboard. They were also told that a larger, second arrow may appear just after the first arrow and, if it does, to ignore the first arrow and press the arrow key that matches the direction of the second arrow. They were not given any information regarding interpretation of the two arrow colors, the frequency, timing, or variability of presentation of the second arrow, the trial response windows, or how to prioritize speed versus accuracy.

**Figure 2:**
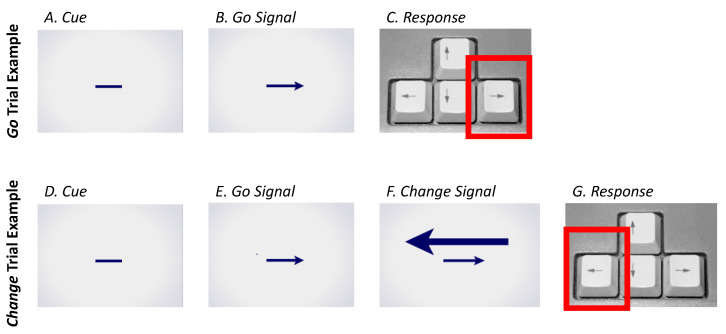
Change Signal task trial examples. Two-thirds of trials in each session were *Go* trials (**A**–**C**), comprised of a *Cue* (**A**) followed by a *Go signal* (left or right arrow; (**B**)), with the subject instructed to make a response according to the *Go signal* arrow (**C**). The remaining one-third of trials in each session were *Change* trials (**D**–**G**), comprised of a *Cue* (**D**) followed by a *Go signal* (left or right arrow; (**E**)), followed by *Change signal* (opposite facing arrow; (**F**)), with the subject instructed to make a response according to the larger *Change signal* arrow (**G**).

Each trial began with the presentation of a line (*Cue*) on the computer screen. After 1 s, this turned into an arrow (*Go* signal) pointing either left or right (equal chance), and subjects were to respond based on the direction of this arrow. However, in 72 trials per session (1/3 of trials, randomly distributed throughout the session), a second, larger, opposite-facing arrow (*Change* signal) was presented at a given delay after the initial *Go* signal (as further described below). On trials with a *Change* signal, subjects were to respond based on this second arrow, rather than the first, original, *Go* arrow (see Figure 2). A blank screen was shown for 500 ms between trials.

Importantly, the delay from the onset of the *Go* stimulus to the presentation of the *Change* stimulus, called the *Change Signal Delay (CSD)*, was dynamically determined based on the subjects’ task performance. The *CSD* became longer following each correct response and shorter following each incorrect response. Because most trials were *Go* trials and subjects were thus primed to respond according to the *Go* signal, a longer delay on *Change* trials would make it more likely that subjects would incorrectly respond to the *Go* instead of the *Change* signal either before or shortly after the appearance of the *Change* signal. Reflecting a previously established positive monotonic relationship between *CSD* length and error rates [32], the length of the *CSD* has a critical influence on expected accuracy on the task, where longer delays should generally result in worse accuracy and shorter delays should generally result in better accuracy.

Each trial used one of two colors for the *Cue/Go/Change* signals; the colors were used to indicate either a *High Error Likelihood* or a *Low Error Likelihood* trial, where error likelihood referred to anticipated rates of incorrect responses as a function of *CSD* dynamics. The colors were randomized for each test session, and the relationship of colors to the *Error Likelihoods* was not announced to the subjects. In both *Error Likelihoods*, the *CSD* was constrained between 20 and 800 ms, and the initial *CSD* value for each test session was 250 ms. The *CSD* then changed in response to the accuracy of the subject’s responses using a staircase-tracking algorithm. A correct response to a *Change* trial lengthened the *CSD* for *Low Error Likelihood* trials by 2 ms and for *High Error Likelihood* trials by 50 ms. In both *Error Likelihoods*, an incorrect response to a *Change* trial shortened the *CSD* by 50 ms. Throughout the session, these dynamics led to shorter *CSDs* for the *Low Error Likelihood* trials and longer *CSDs* for the *High Error Likelihood* trials per the positive monotonic relationship between *CSD* length and error rates [32]. These *CSD* manipulations, originally from Brown and Braver [27], were designed to produce target error rates in *Change* trials of approximately 50% in *High Error Likelihood* trials and 4% in the *Low Error Likelihood* trials under baseline conditions.

The Change Signal task had a response deadline of 1000 ms from the *Go* signal presentation, regardless of whether or not it was followed by a *Change* signal and at what *CSD*. Failures to respond within this deadline were categorized as non-responses and resulted in the same *CSD* shortening as incorrect responses. This forced subjects to limit how long they waited for the possible appearance of a *Change* signal. Thus, the task required subjects to balance the need to make a response quickly with the need to maintain response accuracy given the possibility that the required response would change.

### 2.4. Statistical Analysis

Because response speeds vary in accordance with the *CSD,* analyses of the Change Signal task data focused on the *CSD* length, as a correlate of accuracy, in the *Change* trials, and on the proportion of responses that were incorrect or non-responses in the *Go* and *Change* trials, per phase for each condition (TSD and WRC). The training administration and nighttime administrations during the sleep deprivation period were excluded from analyses, leaving seven daytime sessions per phase in both conditions (numbered Phases 1 through 3 in Figure 1).

For each subject, the median *CSD* length was calculated using the aggregated *Change* trials over the seven test sessions per phase separately for the *High Error Likelihood* and *Low Error Likelihood* trials. The median *CSD* was then analyzed separately for each error likelihood using mixed-effects analysis of variance (ANOVA) with fixed effects for condition (WRC, TSD), phase (1–3), and their interaction, and a random effect over subjects in the intercept. This analysis was repeated for the individuals’ proportion of trials across the seven test sessions per phase that were incorrect responses and that were non-responses in the *Go* and *Change* trials with *Low* or *High Error Likelihood*. To assess the effect of sleep deprivation for each of these analyses, pairwise comparisons (planned contrasts) were then made between Phase 2 in the TSD condition, Phases 1 and 3 in the TSD condition, and Phase 2 in the WRC condition. These analyses were also conducted with an additional covariate for first or second day (a or b) within each phase to distinguish the effects of the first (a) and second (b) days of sleep deprivation. Pairwise comparisons were made between Phase 2 days within each condition and between conditions within each Phase 2 day.

To explore inter-individual differences in Change Signal task performance, the outcome measures were subjected to principal component analysis (PCA), using data from subjects in the TSD condition in Phase 1 and, separately, in Phase 2. Factors were retained based on inspection of the scree plot, and no rotation was applied so as to preserve each factor’s original explained variance. To confirm that results were not an artifact of analyzing random noise, data from Phase 1 (baseline) and Phase 3 (recovery) in the TSD condition (while subjects were rested) were analyzed with a random intercept regression model, after which the intraclass correlation coefficient (ICC) was calculated. The ICC expressed systematic variability between individuals relative to overall variability in the data set, calculated as ICC = ω^2^/(ω^2^ + σ^2^), where ω^2^ represents the variance of the intercept over subjects and σ^2^ represents the residual variance. As such, the ICC measured the stability of inter-individual differences and provided an index of systematic variance in the data as a basis for the PCA.

## 3. Results

### 3.1. Distribution of Change Trial Outcomes by Condition and Phase

Possible outcomes for the *Go* trials were correct responses (i.e., selecting the arrow that matched the *Go* arrow), non-responses (i.e., failing to respond within the response window of 1000 ms from the *Go* arrow presentation), and incorrect responses (i.e., selecting the opposite arrow that did not match the *Go* arrow). The *Change* trials had the same potential correct and non-response outcomes, but incorrect responses could be divided between three types: (1) responding in the direction of the *Go* arrow after presentation of the *Change* arrow (i.e., failure to switch responses), (2) responding in the direction of the *Go* arrow prior to the presentation of the *Change* arrow (i.e., a premature response), or (3) responding in the direction of the *Change* arrow prior to its presentation (i.e., a false start to the future *Change* arrow presentation *or* an incorrect response to the *Go* arrow). However, an actual occurrence of incorrect responses of the second or third type made up less than 3% of *High Error Likelihood* trials and less than 1% of *Low Error Likelihood* trials, so the three potential forms of incorrect responses to *Change* trials were pooled for analyses.

**Figure 3:**
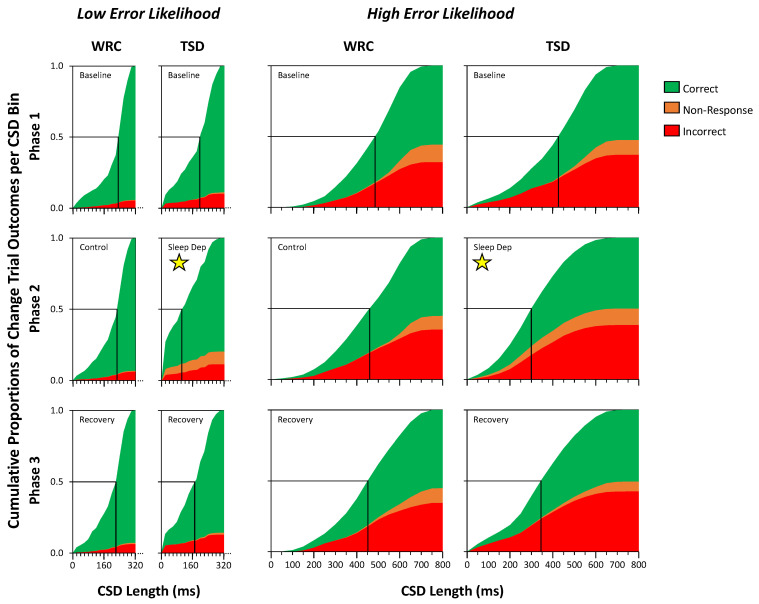
Cumulative proportions of *Change* trial outcomes as a function of *CSD* length, distinguishing *Low* vs. *High Error Likelihood* trials, comparing the WRC and TSD conditions in each phase of the study. *Change* trial outcomes were categorized as correct (green), non-response (orange), or incorrect (red), with the cumulative proportion of each outcome plotted against *CSD* length in 20 ms bins (*Low Error Likelihood* trials, narrow graphs in left two columns) or in 50 ms bins (*High Error Likelihood* trials, wide graphs in right two columns) for Phase 1 (top), Phase 2 (middle), and Phase 3 (bottom). Subjects in the TSD condition were sleep-deprived in Phase 2 (panels marked with a yellow star). The intersection of the horizontal line at 0.5 with the cumulative proportion of trials (marked with a vertical black line) indicates median *CSD* length.

Figure 3 shows the cumulative frequency plots of *Change* trial outcomes as a function of *CSD* length in the *Low Error Likelihood* trials (left two columns) and *High Error Likelihood* trials (right two columns) in each phase of the study for each condition. In the WRC condition, the dynamically changing *CSD* length for the majority of *Change* trials with *Low Error Likelihood* was between about 200 and 300 ms, shifting to between 500 and 800 ms for the majority of *Change* trials with *High Error Likelihood*. The distribution of correct responses (green), non-responses (orange), and incorrect responses (red) stayed reasonably consistent across the three phases for both trial types. The error rate (combined non-responses and incorrect responses) averaged approximately 7% in each phase for the *Low Error Likelihood Change* trials and approximately 45% for the *High Error Likelihood Change* trials. As expected, both non-responses and incorrect responses increased as a function of the *CSD* duration. In the *Low Error Likelihood* trials, errors were almost exclusively incorrect responses and non-responses were rare, whereas in the *High Error Likelihood* trials, incorrect responses were more frequent than non-responses. Performance was stable in the WRC condition across phases.

In the TSD condition, the *Change* trial data were similar, except during Phase 2, when subjects were sleep-deprived (Figure 3 panels marked with a yellow star). In response to changes in response speed and accuracy associated with sleep deprivation, the adaptive algorithm of the task induced a profound shifting of the distribution of *CSD* lengths toward shorter duration, in both the *Low* and *High Error Likelihood* trials. More than 25% of *Change* trials with *Low Error Likelihood* were at the minimum *CSD* length of 20 ms, and the majority of trials had a *CSD* between 20 and 140 ms. The majority of *Change* trials with *High Error Likelihood* had a *CSD* between 200 and 450 ms (e.g., approximately half the duration of the *CSD* for WRC subjects during these trials). Furthermore, Phase 2 in the TSD condition introduced a prominent increase in the proportion of non-responses, particularly in the *Low Error Likelihood* trials, and a change in the distribution of non-responses in the *High Error Likelihood* trials, with non-responses beginning at shorter *CSD* lengths than in Phases 1 or 3. For the *Low Error Likelihood* trials, the error rate (combined non-responses and incorrect responses) averaged approximately 11% in Phase 1, 20% in Phase 2, and 14% in Phase 3, whereas in the *High Error Likelihood* trials, it averaged approximately 50% in each phase. Overall, in the *High Error Likelihood* trials, the adaptive shortening of the *CSD* lengths was such that subjects were able to maintain a relatively low but consistent level of response accuracy, while in the *Low Error Likelihood* trials, response accuracy was higher but the error rate increased during sleep deprivation even with the shortening of the *CSD* lengths.

### 3.2. Analysis of Change Trial Outcomes by Condition and Phase

**Figure 4:**
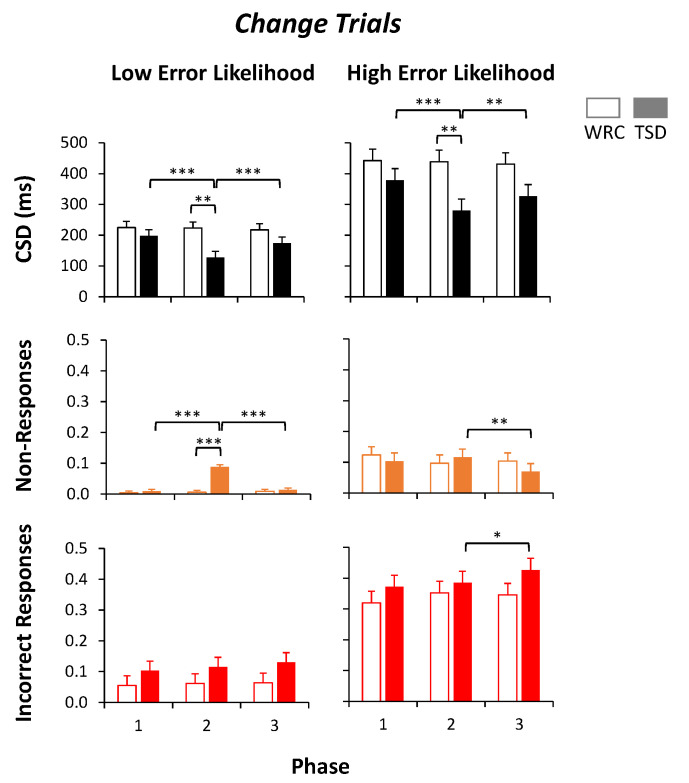
Outcome measures for *Change* trials. The median *CSD* length (top) and proportion of non-responses (middle) and incorrect responses (bottom) for each phase are shown in the *Low Error Likelihood* trials (left column) and in the *High Error Likelihood* trials (right column) for subjects in the well-rested control condition (WRC; white bars) and total sleep deprivation condition (TSD; filled bars). A 0.5 proportion of incorrect responses or non-responses would indicate chance accuracy. *** *p* ≤ 0.001, ** *p* ≤ 0.01, * *p* ≤ 0.05.

Figure 4 quantifies the data of Figure 3 in terms of median *CSD* length, proportion of non-responses, and proportion of incorrect responses in *Change* trials with *Low Error Likelihood* (left) and *High Error Likelihood* (right) across the study phases in each condition. For median *CSD* length in the *Change* trials with *Low Error Likelihood*, there were main effects of condition (*F*_1,48_ = 4.10, *p* = 0.048) and of phase (*F*_2,48_ = 16.92, *p* < 0.001), with a shorter median *CSD* in the TSD condition and in Phase 2, as well as a condition by phase interaction (*F*_2,48_ = 16.29, *p* < 0.001), with the most pronounced decrease in median *CSD* in Phase 2 of the TSD condition (Figure 4, top left). Planned contrasts revealed that the Phase 2 median *CSD* in the TSD condition was significantly shorter than the Phase 2 median *CSD* length in the WRC condition by 95 ± 28 ms (mean ± SE) and the TSD condition median *CSD* lengths in Phase 1 by 71 ± 9 ms and in Phase 3 by 47 ± 9 ms. This can also be seen in Figure 3 (left-hand graphs), where the 0.5 line intersects the cumulative trial distribution at a shorter *CSD* length during Phase 2 in the TSD condition than in the other panels. While Change Signal trial accuracy is a function of *CSD* length as a function of the adaptive algorithm, the presumed balance between *CSD* length and accuracy shifted for sleep-deprived subjects, such that the median *CSD* duration in *Low Error Likelihood* trials was markedly shorter than in Phases 1 and 3 compared to the WRC condition.

Similarly, for median *CSD* length in the *Change* trials with *High Error Likelihood*, there were main effects of condition (*F*_1,48_ = 4.55, *p* = 0.038) and phase (*F*_2,48_ = 8.95, *p* < 0.001), with a shorter median *CSD* in the TSD condition and in Phase 2, as well as a condition by phase interaction (*F*_2,48_ = 7.55, *p* = 0.001), again with the most pronounced decrease in median *CSD* in Phase 2 of the TSD condition (Figure 4, top right). Planned contrasts revealed that the Phase 2 *CSD* in the TSD condition was significantly shorter than the Phase 2 *CSD* in the WRC condition by 158 ± 53 ms and from the TSD condition mean *CSDs* in Phase 1 by 98 ± 17 ms and in Phase 3 by 46 ± 17 ms. This can again also be seen in Figure 3 (right-hand graphs), where the 0.5 line intersects the cumulative trial distribution at a shorter *CSD* length during Phase 2 in the TSD condition than in the other panels. As in the *Low Error Likelihood Change* trials, the relationship between accuracy and *CSD* length was modified by sleep deprivation, such that the median *CSD* duration in *High Error Likelihood Change* trials was markedly shorter than in Phases 1 and 3 and compared to the WRC condition.

In the *Change* trials with *Low Error Likelihood*, the proportion of non-responses showed the main effects of condition (*F*_1,48_ = 27.63, *p* < 0.001), phase (*F*_2,48_ = 33.38, *p* < 0.001), and condition by phase interaction (*F*_2,48_ = 34.04, *p* < 0.001) with a greater proportion of non-responses in the TSD condition, in Phase 2, and particularly in Phase 2 of the TSD condition (Figure 4, middle left). Planned contrasts showed that during Phase 2, subjects in the TSD condition had a 8.3 ± 0.9% higher proportion of non-responses than subjects in the WRC condition. Within the TSD condition, subjects had 8.3 ± 0.8% more non-responses in Phase 2 than in Phase 1 and 7.5 ± 0.8% more non-responses than in Phase 3. However, in the *Change* trials with *Low Error Likelihood*, the proportion of incorrect responses had no significant effects of condition (*p* = 0.20), phase (*p* = 0.16), or condition by phase interaction (*p* = 0.58), showing no effect of sleep deprivation (Figure 4, bottom left). Thus, in *Low Error Likelihood Change* trials, sleep deprivation increased non-responses but not incorrect responses.

In the *Change* trials with *High Error Likelihood,* the proportion of non-responses showed no main effect of condition (*p* = 0.74), but there were trends for phase (*F*_2,48_ = 2.55, *p* = 0.089), with the fewest non-responses in Phase 3, and condition by phase interaction (*F*_2,48_ = 2.53, *p* = 0.090), with fewest non-responses in Phase 3 of the TSD condition (Figure 4, middle right). Planned contrasts showed no difference in the proportion of non-responses during Phase 2 between subjects in the TSD condition and in the WRC condition, or between the TSD condition Phase 2 and Phase 1, although there were 4.7 ± 1.8% more non-responses in Phase 2 compared to Phase 3 of the TSD condition (*p* = 0.010). In addition, in the *Change* trials with *High Error Likelihood,* the proportion of incorrect responses had no significant effect of condition (*p* = 0.28) or condition by phase interaction (*p* = 0.25), but there was a significant effect of phase (*F*_2,48_ = 3.96, *p* = 0.026) with the proportion of incorrect responses increasing across the phases (Figure 4, bottom right). Planned contrasts showed no significant differences between the proportion of incorrect responses during Phase 2 between subjects in the TSD condition and in the WRC condition, or relative to the TSD condition in Phase 1, although there was a small but significant increase in incorrect responses in the TSD condition from Phase 2 to Phase 3 by 4.2 ± 2.0%. Thus, in contrast to the *Low Error Likelihood Change* trials, sleep deprivation did not cause a pronounced increase in non-responses to *High Error Likelihood Change* trials nor did it increase the proportion of non-responses.

### 3.3. Analysis of Go Trial Outcomes by Condition and Phase

**Figure 5:**
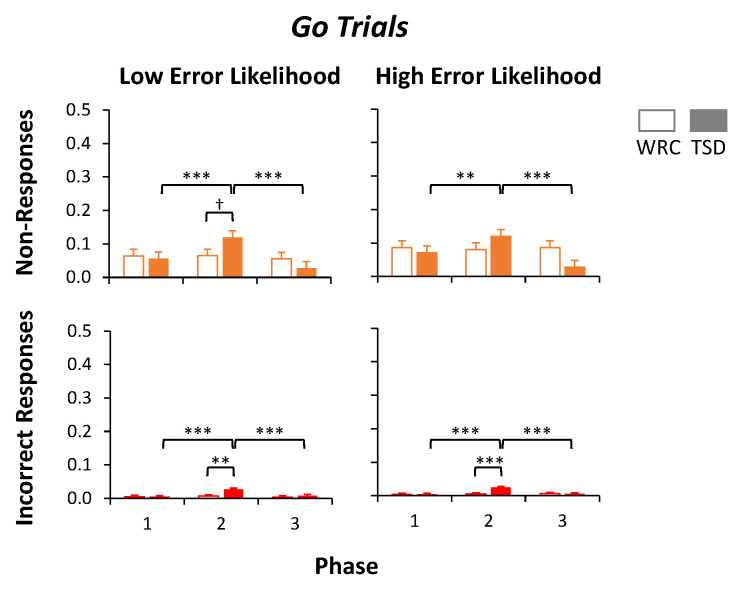
Outcome measures for *Go* trials. The proportion of non-responses (top) and incorrect responses (bottom) for each phase is shown in the *Low Error Likelihood* trials (left column) and in the *High Error Likelihood* trials (right column) for subjects in the well-rested control condition (WRC; white bars) or total sleep deprivation condition (TSD; filled bars). A 0.5 proportion of non-responses or incorrect responses would indicate chance accuracy. *** *p* ≤ 0.001, ** *p* ≤ 0.01, † *p* ≤ 0.10 (trend).

Similarly to Figure 4, Figure 5 shows the proportion of non-responses and proportion of incorrect responses in *Go* trials with *Low Error Likelihood* (left) and *High Error Likelihood* (right) across study phases in each condition. For the proportion of non-responses in *Go* trials with *Low Error Likelihood*, there was no significant effect of condition (*p* = 0.79), but there was a significant effect of phase (*F*_2,48_ = 9.26, *p* < 0.001) and a condition by phase interaction (*F*_2,48_ = 6.61, *p* = 0.003), with the highest proportion of non-responses during Phase 2, especially for TSD subjects (Figure 5, top left). Planned contrasts showed that there was a trend for a greater proportion of non-responses in Phase 2 for subjects in the TSD condition than in the WRC condition by 5.5 ± 2.7%, and there was a significantly greater proportion of non-responses within the TSD condition in Phase 2 than in Phase 1 (by 6.3 ± 1.7%) and in Phase 3 (by 9.1 ± 1.7%). The proportion of incorrect responses in *Go* trials with *Low Error Likelihood* had no significant effect of condition (*p* = 0.12), but there was a significant effect of phase (*F*_2,48_ = 8.14, *p* < 0.001) and a condition by phase interaction (*F*_2,48_ = 5.03, *p* = 0.010), with an increase in the proportion of incorrect responses during Phase 2, and especially in Phase 2 for TSD subjects (Figure 5, bottom left). While the proportion of incorrect responses in the *Go* trials with *Low Error Likelihood* averaged less than 3% across all phases for both conditions, the planned contrasts revealed a small but significant increase (1.9 ± 0.6%) in incorrect responses in Phase 2 for the TSD condition compared to the WRC condition as well as in the TSD condition during Phase 2 compared to Phase 1 (by 2.2 ± 0.5%) and to Phase 3 (by 1.8 ± 0.5%). Thus, in *Go* trials with *Low Error Likelihood,* sleep deprivation increased the proportions of both non-responses and incorrect responses, although the overall proportion of incorrect responses was still low.

The proportion of non-responses in *Go* trials with *High error Likelihood* had no significant effect of condition (*p* = 0.64), but there was a significant effect of phase (*F*_2,48_ = 5.45, *p* = 0.007) and condition by phase interaction (*F*_2,48_ = 7.01, *p* = 0.002), with the largest proportion of non-responses in Phase 2, especially in TSD subjects (Figure 5, top right). Planned contrasts showed that there was no difference between the proportion of non-responses during Phase 2 between subjects in the TSD condition and in the WRC condition, although the proportion of non-responses was higher in the TSD condition during Phase 2 than Phase 1 (by 4.9 ± 1.9%; *p* = 0.010) and Phase 3 (by 9.2 ± 1.9%; *p* < 0.001). The proportion of incorrect responses in *Go* trials with *High Error Likelihood* had no significant effect of condition (*p* = 0.12), but it did have a significant effect of phase (*F*_2,48_ = 11.69, *p* < 0.001) and a condition by phase interaction (*F*_2,48_ = 9.95, *p* < 0.001) with the largest proportion of incorrect responses in Phase 2, especially in the TSD condition (Figure 5, bottom right). As in the *Low Error Likelihood* trials, *Go* trials with *High Error Likelihood* had a low proportion of trials with incorrect responses throughout the phases and conditions overall (all means < 3%). Planned contrasts revealed a significantly higher proportion of incorrect responses during Phase 2 in the TSD condition than in the WRC condition (by 1.9 ± 0.5%; *p* < 0.001) and within the TSD condition in Phase 2 compared to Phase 1 (by 2.1 ± 0.4%; *p* < 0.001) and to Phase 3 (by 1.9 ± 0.4%; *p* < 0.001). Similarly to the *Go* trials with *Low Error Likelihood,* sleep deprivation increased the proportions of both non-responses and incorrect responses in *Go* trials with *High Error Likelihood,* albeit that the overall proportion of incorrect responses remained low.

### 3.4. Analyses by Day of Sleep Deprivation

**Figure 6:**
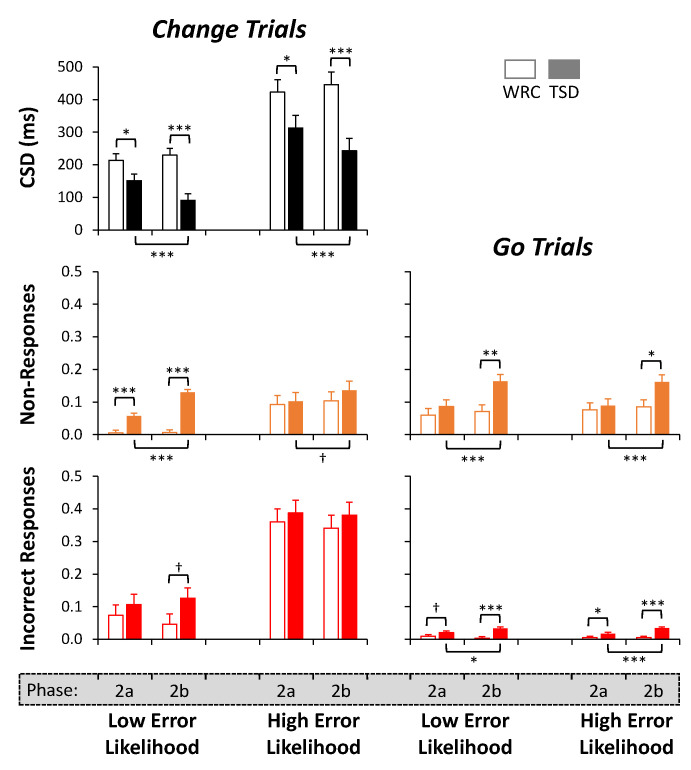
Outcome measures by day (a versus b) of Phase 2. The median *CSD* length (top left) and the proportion of non-responses (middle panels) and incorrect responses (bottom panels) in the *Change* trials (left panels) and *Go* trials (right panels) are displayed. Each panel shows data from subjects in the well-rested control condition (WRC; white bars) and total sleep deprivation condition (TSD; filled bars) on Phases 2a and 2b (i.e., the first and second days of Phase 2). The left four bars in each panel reflect the *Low Error Likelihood* trials, and the right four bars reflect the *High Error Likelihood* trials. Significant differences in pairwise comparisons between conditions for each day are shown with brackets above the bars. Significant differences between days within the TSD condition are shown with brackets below the bars. Note that there were no significant differences between Phase 2a and 2b for any outcome variable in the WRC condition. *** *p* ≤ 0.001, ** *p* ≤ 0.01, * *p* ≤ 0.05, † *p* ≤ 0.10 (trend).

To examine whether the duration of sleep deprivation influenced the magnitude of the performance changes, each of the analyses for median *CSD*, proportion of non-responses, and proportion of incorrect responses were repeated with an additional covariate of day (a or b) within each phase. The mean (±SE) of the Phase 2a and 2b outcomes in the WRC and TSD conditions are plotted in Figure 6. Pairwise comparisons were made between days (Phases 2a and 2b) within each condition and between conditions within each day.

Within Phase 2a, the median *CSD* length was significantly shorter in the TSD condition than in the WRC condition for *Change* trials with *Low Error Likelihood* (*p* = 0.036) and with *High Error Likelihood* (*p* = 0.045). The TSD subjects also produced significantly greater proportions of non-responses to *Change* trials with *Low Error Likelihood* (*p* < 0.001) and incorrect responses to *Go* trials with *High Error Likelihood* (*p* = 0.029). Furthermore, there was a trend for a greater proportion of incorrect responses to *Go* trials with *Low Error Likelihood* in the TSD condition compared to the WRC condition (*p* = 0.098).

Within Phase 2b, the median *CSD* length was again significantly shorter in the TSD condition than in the WRC condition for *Change* trials with *Low Error Likelihood* (*p* < 0.001) and with *High Error Likelihood* (*p* < 0.001). There was a significant difference in *Change* trial outcomes, with the TSD subjects producing a greater proportion of non-responses (*p* < 0.001) with a trend for a greater proportion of incorrect responses to trials with *Low Error Likelihood* (*p* = 0.081). For the *Go* trials of Phase 2b, the TSD subjects produced a greater proportion of non-responses to trials with *Low Error Likelihood* (*p* = 0.002) and *High Error Likelihood* (*p* = 0.014), and there was a greater proportion of incorrect responses to trials with *Low Error Likelihood* (*p* < 0.001) and *High Error Likelihood* (*p* < 0.001).

There were no significant differences in any of the outcome measures between Phase 2a and 2b within the WRC condition. By contrast, the TSD condition showed effects of sleep deprivation duration (day 2 versus day 1) on the median *CSD* length in *Change* trials with *Low Error Likelihood* (*p* < 0.001) and *High Error Likelihood* (*p* < 0.001). There were effects of sleep deprivation duration for the proportion of non-responses to *Change* trials with *High Error Likelihood* (*p* < 0.001) and the proportion of non-responses to *Go* trials with *Low Error Likelihood* (*p* < 0.001) and *High Error Likelihood* (*p* < 0.001) as well, with a trend for an effect in *Change* trials with *Low Error Likelihood* (*p* = 0.092). In addition, within the TSD condition, there was an effect of sleep deprivation duration on the proportion of incorrect responses to *Go* trials with *Low Error Likelihood* (*p* = 0.019) and *High Error Likelihood* (*p* < 0.001). Thus, the impact of sleep deprivation on Change Signal task performance revealed multiple dose–response effects.

### 3.5. Individual Differences in Change Signal Performance by Phase in the TSD Condition

To explore distinct aspects of the impact of sleep deprivation on Change Signal task performance, we conducted a PCA using the TSD subject data in Phases 1 and 2. Each analysis included 10 variables (five variables for each of the *Low Error Likelihood* and *High Error Likelihood* trials): the median *CSD* length and the mean proportions of incorrect responses and non-responses to *Change* and *Go* trials. For Phase 1 (baseline), the scree plot pointed to just one dominant factor, which alone explained 73.3% of the variance. For Phase 2 (sleep deprivation), however, a second dominant factor emerged, with Factor 1 explaining 47.2% of the variance and Factor 2 explaining 40.8% (for a combined 88.0% of variance explained). As shown in Table 1, the proportion of incorrect responses to *Go* trials with *High Error Likelihood* and both *Go* and *Change* trials with *Low Error Likelihood*, as well as the median *CSD* for both *Low* and *High Error Likelihood* trials, loaded predominantly on the first factor. All four proportions of non-response outcomes (in the *Go* and *Change* trials with both *Low* and *High Error Likelihood*) and the proportion of incorrect responses to *Change* trials with *High Error Likelihood* loaded primarily on the second factor. These results indicate that inter-individual differences in the impact of sleep deprivation on Change Signal performance were not one-dimensional but rather showed two distinct characteristics that varied across individuals independently.

To confirm that the emergence of only one prominent factor at baseline was not merely due to a lack of non-random variance in the data, we calculated the ICC for each of the 10 Change Signal outcome variables using data from the TSD condition while subjects were rested during Phase 1 (baseline) and Phase 3 (recovery). The ICC scores all indicated moderate, substantial, or almost perfect stability of inter-individual differences [33], with values ranging from 0.576 (incorrect responses on the *Go* trials with *High Error Likelihood*) to 0.958 (incorrect responses on the *Change* trials with *Low Error Likelihood*); see Table 2. As such, it appeared that the one-factor PCA solution at baseline was meaningful and not an artifact due to analyzing random noise.

## 4. Discussion

The Change Signal task is highly dynamic and, arguably, complex. Performance on the task and the effects of sleep deprivation thereon are not well captured by global outcome measures (e.g., response times, accuracy) nor is the task readily suitable for the decomposition of performance into dissociable aspects of cognition [25]. A key facet of the task is the dynamic *CSD* length, which varies based on *Change* trial accuracy such that it is shortened in response to an error (making the task easier for the next *Change* trial) or longer in response to a correct response (making the task more error-prone for the next *Change* trial). Furthermore, there is a 1000 ms response window beginning with the presentation of the *Go* stimulus for all trials; thus, a longer *CSD* leaves a shorter opportunity to respond after the appearance of a *Change* signal. These dynamic changes vary between *Low* and *High Error Likelihood* trials and serve to maintain specific levels of accuracy, at least under rested conditions. As such, neither response accuracy nor response speed are informative outcomes on the task, but *CSD* length can be interpreted as a proxy of error-proneness. Additionally, the proportions of non-responses and incorrect responses shed light on the types of errors being produced.

### 4.1. Well-Rested Performance

Performance in the WRC condition was generally stable across all three phases. The median *CSD* length was approximately 220 ms in trials with *Low Error Likelihood* and approximately twice as long in trials with *High Error Likelihood*. These *CSD* lengths produced overall error rates of approximately 7% for each phase in *Change* trials with *Low Error Likelihood* and approximately 45% for each phase in *Change* trials with *High Error Likelihood*, which is in agreement with the differentiation of these trial types as intended with the task design. In the *Low Error Likelihood Change* trials, the errors were almost exclusively incorrect responses. In the *High Error Likelihood Change* trials, where errors were more common and *CSDs* tended to be longer, the correspondingly shorter opportunity to respond that remained after a *Change* signal increased the propensity for non-response errors. There were approximately 1:3 non-responses to incorrect responses in the *High Error Likelihood* trials.

The mental demand of the task may in part be related to the balance between weighing how long to wait for a *Change* signal to be presented, which may or may not occur on a given trial, against when to go ahead with a response in order to avoid the trial timing out and resulting in a non-response. This may be akin to tasks with variable response deadlines such as the Psychomotor Vigilance Test [34], for which the mental demand has been attributed to the requirement to maintain a tightly calibrated response criterion [35] as needed to balance between errors of omission (waiting too long) and errors of commission (responding too early) [21]. On the Change Signal task, non-responses (i.e., waiting too long) were a function of time pressure or possibly of failure to adequately manage the time pressure. In the WRC condition, the lowest occurrence of non-responses was in *Change* trials with *Low Error Likelihood.* In such trials, since the *Change* has been presented, there was no longer a need to balance waiting versus responding and, furthermore, time pressure was lowest since the shorter *CSD* left more time to make a response after the presentation of a *Change* signal before the trial would time out. By contrast, in the *Change* trials with *High Error Likelihood*, the longer *CSDs* left less time to make a response after the presentation of a *Change* signal, and non-responses were more common.

In the *Go* trials (with both *Low* and *High Error Likelihood*), non-responses were more frequent, suggesting a failure to balance between responding versus waiting for a *Change* signal that was not going to be presented. Even so, the *Go* trials in the WRC condition revealed that the essence of the task was not difficult to complete, as errors (combined incorrect and non-responses) made up less than 10% of *Go* trials for WRC subjects in each phase. Furthermore, subjects favored accuracy over speed such that incorrect responses to *Go* trials were almost non-existent (less than 0.5% of *Go* trials in the WRC condition). That is, WRC subjects were able to accurately encode the *Go* arrows and execute the corresponding response with near-perfect accuracy.

However, incorrect responses to *Change* trials were more prominent, especially in *High Error Likelihood* trials. Judging by the lack of premature responses in *Change* trials as well as the lack of incorrect responses in *Go* trials, these incorrect responses were not a reflection of impulsivity-driven responding prior to the *Change* signal. Rather, they typically occurred after the *Change* signal had been presented and likely reflected the burden of needing to inhibit (or release) the planned *Go* response, or stop a *Go* response that had already been initiated, in order to switch to the *Change* response. Previous studies using go/no-go tasks have shown that time pressure impairs inhibition accuracy [36]. Consistent with this notion, in the *High Error Likelihood Change* trials, where time pressure was more pronounced than in the *Low Error Likelihood Change* trials, there was not only an increase in the number of non-responses but also a greater proportion of incorrect responses.

### 4.2. Effects of Sleep Deprivation

During sleep deprivation (i.e., Phase 2 in the TSD condition), the adaptive *CSD* feature of the Change Signal task no longer had the intended effect of clamping accuracy rates. Although the median *CSD* length differed between the *Low* and *High Error Likelihood* trials and the error rate in the *High Error Likelihood* condition stayed reasonably stable compared to baseline, the error rate in the *Low Error Likelihood Change* trials was nearly twice as high as during baseline. Because of the interconnectedness of the task metrics (e.g., *CSD* length relating to the error rate), the underlying causes of some performance changes during sleep deprivation cannot be pinpointed precisely. Nonetheless, several noteworthy performance changes and potential reasons for such changes emerged.

One of the most striking changes during sleep deprivation in both the *Low* and *High Error Likelihood Change* trials was in the *CSD* length. For the *Low Error Likelihood* trials, the median *CSD* during Phase 2 of the TSD condition (i.e., during sleep deprivation) was approximately 70 ms shorter than during Phase 1 (baseline); for the *High Error Likelihood* trials, the difference was more than 150 ms. Even though the preponderance of shorter *CSDs* left longer response windows on *Change* trials, the proportion of non-responses increased in the *Low Error Likelihood* trials during sleep deprivation. This was likely at least partially due to the hallmark “wake state instability” effect of sleep deprivation on cognitive processing [21,23,37], which involves a rightward skewing of reaction time distributions [38]. Here, this skewing would have pushed a greater proportion of response times out of the fixed response window, resulting in non-responses. The skewing of the reaction time distribution would presumably have influenced performance on the *High Error Likelihood Change* trials as well. On the latter, however, there was a more dramatic shift to shorter *CSDs*, which would have counteracted that effect, and non-responses on these trials were already more frequent at baseline—the net effect appeared to be that while sleep-deprived, subjects kept the non-response rate on the *High Error Likelihood* trials near baseline levels.

The proportion of errors (combined incorrect and non-responses) in *Change* trials varied between *Low* and *High Error Likelihood* in the WRC condition, which was presumably due to the difference in *CSD* durations. However, even though the *CSD* was markedly reduced during sleep deprivation, there was no commensurate reduction in errors. Rather, even with more time between the shorter *CSD* and the end of the trial to inhibit the initial response and switch to the *Change* response, the proportion of errors stayed approximately even for the *High Error Likelihood* trials, whereas it increased in the *Low Error Likelihood* trials (specifically through non-responses). This differential effect of sleep deprivation on errors during *Change* trials likely reflected a floor effect in the *Low Error Likelihood* trials, as the *CSD* was at the minimum 20 ms for more than a quarter of such trials during sleep deprivation. Thus, sleep deprivation likely impaired the ability to inhibit the *Go* response—or release a response that had already been initiated—and switch to the *Change* response, which is consistent with previous reports that the ability to withhold responses on a go/no-go task is impaired by sleep loss [39].

An increase in non-responses during sleep deprivation was seen on the *Go* trials, which was likely due in part to the same underlying mechanisms as in the *Change* trials. However, an additional variable may be in effect in the *Go* trials, as unlike the *Change* trials, *Go* trials have the added component of balancing how long to wait for a potential *Change* to occur before making a response. Given that the response window was always 1000 ms from the onset of the *Go* trial, the increase in non-responses to *Go* trials could have been in part due to changes in time estimation [40]. That is, subjects may have been less able to effectively gauge how quickly they needed to respond within a trial. Additionally, there was a small but statistically significant increase in the proportion of incorrect responses on the *Go* trials. This may reflect a struggle to maintain a consistent speed/accuracy balance under sleep deprivation. Perhaps in response to the increase in time-outs (i.e., errors from favoring accuracy over speed), subjects exerted a compensatory effort to respond quickly, which then resulted in occasional incorrect responses (i.e., errors from favoring speed over accuracy). This is consistent with the idea that in tasks with a strong vigilant attention component, false starts may increase during sleep deprivation as a reflection of compensatory effort [21].

The changes in performance in the TSD condition were already present during the first day of sleep deprivation (i.e., Phase 2a). On the first day of sleep deprivation, there were shorter median *CSD* lengths in the trials with *Low Error Likelihood* and *High Error Likelihood* compared to the WRC condition. Furthermore, there was a greater proportion of non-responses to *Change* trials with *Low Error Likelihood* and a greater proportion of incorrect responses to *Go* trials with *High Error Likelihood.* There were dose–response effects, such that performance changed from the first to the second day of sleep deprivation on most outcome measures (see Figure 6). While the overall intervention of 62 h of continuous wakefulness may be more extreme than is typically encountered in everyday life, these observations demonstrate that performance was already changing beyond what the task was able to adapt to at more common levels of sleep deprivation (i.e., within the first day of sleep deprivation). Furthermore, as sleep deprivation continued, the effects were amplified, and there was no evidence of a ceiling effect.

### 4.3. Distinct Aspects of Performance

An examination of inter-individual differences in Change Signal task performance revealed a potentially important effect of sleep deprivation. That is, while the various outcome measures of the task clustered along a single dimension when subjects were well-rested, a second dimension emerged when subjects were sleep-deprived. Sleep deprivation is a powerful paradigm for probing cognitive mechanisms underlying performance impairment [2], and as such, it is no surprise that sleep deprivation would reveal distinct facets of Change Signal task performance that are not observed under rested conditions. However, unlike in other studies where this phenomenon has been observed [41,42,43], here, it is not readily apparent what differentiates the two dimensions.

One aspect of performance on the Change Signal task that may vary between subjects is the speed/accuracy trade-off [44]. In the *Go* trials, well-rested subjects appeared to favor accuracy over speed, such that there were occasional non-responses (i.e., time-outs), but incorrect responses were quite rare. During sleep deprivation, this balance may have shifted, as accuracy on the *Go* trials was not maintained at the same level as when rested. In addition to trait differences in the degree to which individuals are naturally inclined to favor speed over accuracy or vice versa [45], there may have been inter-individual differences in how the speed/accuracy trade-off was altered during sleep deprivation, which could explain the emergence of a second dimension of performance impairment.

The contribution of some other elements of cognition to the clustering of performance outcomes in two dimensions during sleep deprivation can be plausibly ruled out. For instance, the lack of an effect of sleep deprivation on incorrect responses to the *Change* trials suggests four things. First, this result is congruent with previous findings that sleep deprivation does not significantly interfere with the encoding of stimuli [5]. Second, it is consistent with a recent report that sleep deprivation does not significantly degrade the planning and execution of motor actions [46]. Third, although sleep-deprived subjects had more time to process the stimuli given shorter *CSDs*, reflecting a likely speed–accuracy trade-off, sleep deprivation did not seem to worsen their ability to resolve proactive interference from a planned *Go* response that was to be replaced by a *Change* response [46]. Last, the lack of a sleep deprivation effect on incorrect responses is consistent with the notion that sleep deprivation does *not* necessarily promote impulsivity, as has been pointed out before [47]. These considerations notwithstanding, what drives the emergence of a second dimension of Change Signal performance during sleep deprivation remains an outstanding question.

## 5. Conclusions

Sleep deprivation altered performance on the Change Signal task such that the median length of *CSDs* was shorter in both *Low Error Likelihood* and *High Error Likelihood Change* trials. This kept the overall error rate at baseline levels for the *High Error Likelihood Change* trials, but for the *Low Error Likelihood Change* trials, the error rate increased to approximately twice the baseline rate. Response speeds showed changes characteristic of wake-state instability, with increasing variability and a likely skewing of the response time distribution such that there were more non-responses in the *Go* trials and in the *Low Error Likelihood Change* trials. The same also presumably affected the *High Error Likelihood Change* trials, but the greater shift in *CSD* length and the higher non-response rate at baseline may have counteracted this. Furthermore, whereas variance in Change Signal task outcomes clustered along a single dimension at baseline, a second dimension emerged during sleep deprivation, indicating that sleep deprivation brings out two distinct facets of inter-individual variability in performance on the task.

In military missions, emergency response, health care, and other operational settings that commonly involve sleep deprivation, individuals are often also faced with balancing competing task demands, such as maintaining performance accuracy while negotiating time pressure. Analogously, in the present Change Signal task, the goal was to respond both accurately (i.e., responding correctly based on the *Go* or *Change* arrows) and fast (i.e., under time pressure). Subjects in the WRC condition were able to reasonably handle both demands; both incorrect responses and time-outs were rare. By contrast, subjects in the TSD condition experienced increased errors during Phase 2 (i.e., while sleep deprived) both in the *Go* trials and the *Low Error Likelihood Change* trials. Notably, time pressure was lessened during TSD, as the *CSD* was significantly shorter, leaving more time to respond within the 1000 ms response window. Yet, even with less time pressure, the error rate for *Low Error Likelihood* trials increased during TSD, and it exceeded the target rate aimed for through the parameters of the adaptive *CSD* dynamics. This change in response behavior during TSD is reminiscent of the shift in speed/accuracy trade-off that sleep-deprived people have been found to adopt in other tasks (e.g., [48]) despite there being no externally imposed change in demand characteristics. Possibly related cognitive tasks that require significant attentional control to perform well seem to be especially sensitive to sleep loss when time pressure is involved [20,35,47]. Determining whether the interplay of sleep loss, time pressure, and attentional control is at the nexus of vulnerability to performance impairment could have important operational implications.

As they relate to augmented cognition, our results suggest that interventions found to protect performance accuracy under well-rested conditions, such as adaptive task pacing, should not automatically be assumed to be equally effective when people are sleep deprived. This has real-world relevance in a range of safety-sensitive and time-critical scenarios. For instance, when military personnel conduct time-sensitive analyses of land, sea floor, or air to locate and identify threats, adaptive pacing may substantially improve accuracy under well-rested conditions but maybe not as much when sleep-deprived. Similarly, automatic target recognition (ATR) algorithms may enhance both speed and accuracy of target or threat detection [49] by focusing the task on review of automatically detected objects and confirming or rejecting them as potential targets or threats [50]. However, if an operator is conducting an ATR-assisted task while fatigued due to sleep loss, false positives and/or false negatives may be increased compared to when well-rested [51]. A number of related questions and concerns will need to be confronted in current and planned automation efforts for automobile driving where, depending on the level of automation, fatigued drivers may not benefit as much (or may even incur greater risk) compared to their well-rested counterparts [52]. Further research is needed to investigate how various approaches to automation and augmented cognition hold up under conditions of sleep deprivation, particularly in safety-critical tasks with an element of time pressure.

## Figures and Tables

**Table 1:** PCA factor loadings for Change Signal outcomes in the TSD condition during Phase 2 (sleep deprivation). Each outcome measure has the higher factor loading shaded in gray to indicate which primarily load to Factor 1 or Factor 2.

	Factor 1	Factor 2
Incorrect responses *High Error Likelihood Go* trials	0.908	−0.109
Incorrect responses, *Low Error Likelihood Change* trials	0.881	−0.382
Incorrect responses, *Low Error Likelihood Go* trials	0.867	−0.244
Median *CSD,* *Low Error Likelihood Change* trials	−0.830	0.183
Median *CSD,* *High Error Likelihood Change* trials	−0.899	0.130
Non-responses, *High Error Likelihood Change* trials	0.172	0.968
Non-responses, *Low Error Likelihood Go* trials	0.357	0.922
Non-responses, *High Error Likelihood Go* trials	0.399	0.880
Non-responses, *Low Error Likelihood Change* trials	0.636	0.663
Incorrect responses, *High Error Likelihood Change* trials	0.393	−0.898

**Table 2:** ICCs for Change Signal outcomes in the TSD condition during Phases 1 and 3 (baseline and recovery) ordered by ICC magnitude.

	ICC
Incorrect responses, *Low Error Likelihood Change* trials	0.958
Median *CSD,* *Low Error Likelihood Change* trials	0.937
Incorrect responses, *High Error Likelihood Change* trials	0.929
Median *CSD,* *High Error Likelihood Change* trials	0.917
Non-responses, *Low Error Likelihood Go* trials	0.808
Non-responses, *High Error Likelihood Change* trials	0.808
Non-responses, *Low Error Likelihood Change* trials	0.764
Non-responses, *High Error Likelihood Go* trials	0.695
Incorrect responses, *Low Error Likelihood Go* trials	0.653
Incorrect responses *High Error Likelihood Go* trials	0.576

## Data Availability

Upon reasonable request to K.A.H., the data can be shared with researchers.
